# INPP4B is upregulated and functions as an oncogenic driver through SGK3 in a subset of melanomas

**DOI:** 10.18632/oncotarget.5359

**Published:** 2015-11-09

**Authors:** Meng Na Chi, Su Tang Guo, James S. Wilmott, Xiang Yun Guo, Xu Guang Yan, Chun Yan Wang, Xiao Ying Liu, Lei Jin, Hsin-Yi Tseng, Tao Liu, Amanda Croft, Hubert Hondermarck, Richard A. Scolyer, Chen Chen Jiang, Xu Dong Zhang

**Affiliations:** ^1^ School of Medicine and Public Health, The University of Newcastle, NSW 2308, Australia; ^2^ School of Biomedical Sciences and Pharmacy, The University of Newcastle, NSW 2308, Australia; ^3^ Department of Molecular Biology, Shanxi Cancer Hospital and Institute, Affiliated Hospital of Shanxi Medical University, Taiyuan, Shanxi 030013, China; ^4^ Discipline of Pathology, The University of Sydney, and Tissue Pathology and Diagnostic Oncology, Royal Prince Alfred Hospital, Sydney, NSW 2006, Australia; ^5^ Children's Cancer Institute Australia for Medical Research, The University of New South Wales, Sydney, NSW 2052, Australia

**Keywords:** INPP4B, SGK3, melanoma, miRNA-494, miRNA-599

## Abstract

Inositol polyphosphate 4-phosphatase type II (INPP4B) negatively regulates PI3K/Akt signalling and has a tumour suppressive role in some types of cancers. However, we have found that it is upregulated in a subset of melanomas. Here we report that INPP4B can function as an oncogenic driver through activation of serum- and glucocorticoid-regulated kinase 3 (SGK3) in melanoma. While INPP4B knockdown inhibited melanoma cell proliferation and retarded melanoma xenograft growth, overexpression of INPP4B enhanced melanoma cell and melanocyte proliferation and triggered anchorage-independent growth of melanocytes. Noticeably, INPP4B-mediated melanoma cell proliferation was not related to activation of Akt, but was mediated by SGK3. Upregulation of INPP4B in melanoma cells was associated with loss of miRNA (miR)-494 and/or miR-599 due to gene copy number reduction. Indeed, overexpression of miR-494 or miR-599 downregulated INPP4B, reduced SGK3 activation, and inhibited melanoma cell proliferation, whereas introduction of anti-miR-494 or anti-miR-599 upregulated INPP4B, enhanced SGK3 activation, and promoted melanoma cell proliferation. Collectively, these results identify upregulation of INPP4B as an oncogenic mechanism through activation of SGK3 in a subset of melanomas, with implications for targeting INPP4B and restoring miR-494 and miR-599 as novel approaches in the treatment of melanomas with high INPP4B expression.

## INTRODUCTION

Aberrant activation of survival-signalling pathways causes uncontrolled cell proliferation and resistance to apoptosis, and plays an important role in cancer development, progression, and resistance to treatment [1, 2]. In melanoma, identification of activating mutations in BRAF as the major cause of constitutive activation of the mitogen activated protein kinase (MAPK) pathway has led to successful development of mutant BRAF-specific inhibitors in the treatment of the disease [3–6]. However, primary and acquired resistance, which is commonly associated with activation of other survival pathways, in particular, the phosphatidylinositol 3-kinase (PI3K) signalling pathway, remains a major obstacle in the quest for curative treatment [7–10].

PI3K signalling is initiated with the engagement of extracellular growth factors to receptor tyrosine kinases (RTKs). This results in recruitment of PI3K to plasma membrane-anchored receptors where it is activated, leading to increases in the production of phosphatidylinositol(3,4) bisphosphate (PI(3,4)P_2_) and phosphatidylinositol(3,4,5) trisphosphate (PI(3,4,5)P_3_), which in turn bind to and activate multiple downstream effectors [11–13]. Among them is Akt that is activated by two phosphorylation events at Thr308 and Ser473 involving phosphoinositide-dependent kinase 1 (PDK1) and the mammalian target of rapamycin complex 2 (mTORC2) complex, respectively [14, 15]. Activated Akt then phosphorylates a large array of substrates to promote cell survival and proliferation [16, 17].

PI3K can also drive oncogenic signalling independently of Akt through activation of serum- and glucocorticoid-regulated kinase (SGK), another family of serine/threonine kinases consisting of 3 isoforms, SGK1, SGK2, and SGK3 [18]. SGKs are highly homologous to and share substrate specificity with Akt, and are also activated by PI3K involving PDK1 and mTORC2 [19, 20]. Indeed, similar to Akt, SGK1 and SGK3 are involved in the pathogenesis of various types of cancers [21, 22]. Of particular interest, SGK3 has recently been reported to contribute to the growth of mutant BRAF melanomas [23]. Another unique feature of SGK3 is that it contains an N-terminal PX domain that enables its binding to PI(3)P, thus targeting it to early endosomes where it is fully activated [18, 20]. Although SGK3 can be phosphorylated at both Thr320 and Ser486, its full activation requires phosphorylation at Thr320 [20, 24].

Activation of PI3K signalling is negatively regulated by three classes of inositol polyphosphate phosphatases [25–27]. The inositol polyphosphate 3-phosphatase (3-phosphatase) PTEN dephosphorylates the 3-position of PI(3,4,5)P_3_ to generate PI(4,5)P_2_ [28, 29], whereas 5-phosphatases, such as Src homology 2-containing inositol 5- phosphatase (SHIP) and phosphatidylinositol 4,5-Bisphosphate 5-Phosphatase (PIB5PA)/proline-rich inositol polyphosphate phosphatase (PIPP) dephosphorylate the 5-position to produce PI(3,4)P_2_ [30, 31]. The latter is in turn subjected to dephosphorylation by inositol polyphosphate 4-phosphatase type I (INPP4A) and type II (INPP4B) at the 4-position to generate PI(3)P, thus terminating PI3K signalling [25, 26, 32].

While PTEN and some 5-phosphatases such as SHIP2 and PIB5PA are tumour suppressors, the 4-phosphatase INPP4B also plays a tumour suppressive role in variety types of cancers [25, 26, 30, 31]. In particular, a recent study reported a tumour suppressive function for INPP4B in melanocytic neoplasm that was primarily mediated by a small isoform of the protein [33]. Nevertheless, INPP4B-dependent activation of SGK3 drives tumourigenesis in a subset of breast cancers with low Akt [34]. Moreover, INPP4B is associated with chemoresistance and poor outcome of patients with acute myeloid leukaemia (AML) [35, 36]. It seems that the role of INPP4B in the pathogenesis of cancer is highly cell type- and context-dependent. Indeed, we have found that INPP4B functions as an oncogenic driver in human melanoma. In this report, we provide evidence that INPP4B is upregulated in a subset of melanomas and plays a role in melanoma cell proliferation independently of Akt through activating PI3K/SGK3 signalling. Moreover, we show that the increase in INPP4B in melanoma is due to loss of microRNA-494 (miR-494) and/or miR-599 as a result of copy number reduction of chromosome segments where they are respectively located.

## RESULTS

### INPP4B is upregulated in a subset of melanomas

**Figure 1:**
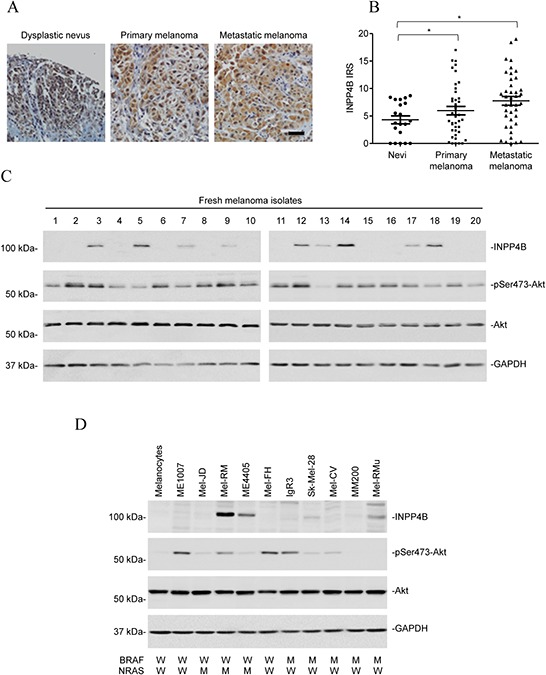
INPP4B is commonly upregulated in melanomas **A.** Representative microphotographs of IHC staining of INPP4B in melanocytic tumour tissue arrays. Scale bar, 100 μm. **B.** Comparison of INPP4B expression among nevi (*n* = 20), primary melanomas (*n* = 40), and metastatic melanomas (*n* = 40) determined by IHC staining. Data shown are mean immunoreactive score (IRS) ± SEM. *P < 0.05, Kruskal-Wallis test. **C.** Whole cell lysates from a panel of fresh metastatic melanoma isolates were subjected to Western blot analysis of INPP4B, phosphorylated Akt (pSer473-Akt), Akt and GAPDH (as a loading control). The data shown are representative of three individual experiments. **D.** Whole cell lysates from pooled melanocytes of three different lines (HEMa-LP, HEMn-MP and HEMn-DP) and melanoma cell lines were subjected to Western blot analysis of INPP4B, phosphorylated Akt (pSer473-Akt), Akt and GAPDH (as a loading control). The data shown are representative of three individual experiments.

We examined the expression of INPP4B in relation to melanoma development and progression by immunohistochemistry (IHC) in tissue microarrays (TMAs) constructed from 100 formalin-fixed paraffin-embedded (FFPE) melanocytic tumours using an antibody against INPP4B that had been used for similar studies in other types of tissues (Supplementary Table 1) [26, 32]. Although INPP4B was commonly detected with weak to moderate staining in nevi, its expression was noticeably elevated in a subset of melanomas (Figures 1A and 1B). However, there was no significant difference in INPP4B levels between thin and thick primary melanomas, or between primary and metastatic melanomas (Figure 1B and Supplementary Table 1), suggesting that INPP4B is upregulated at early stages during melanoma development in a subgroup of patients. The increased expression of INPP4B in a subset of melanomas was confirmed in 20 fresh metastatic melanoma isolates using Western blot analysis (Figure 1C). Of note, there was no noticeable relationship between the levels of INPP4B and activation (phosphorylation) of Akt.

We also examined the expression of INPP4B in a panel of melanoma cell lines compared with pooled melanocytes of three different lines (HEMa-LP, HEMn-MP and HEMn-DP; pooled normal melanocytes were used to simplify analysis, as these melanocyte lines similarly did not express detectable INPP4B by immunoblotting (Supplementary Figure 1)). The melanoma cell lines had various statuses of the most common mutations in *BRAF* (BRAF^V600E^) and *NRAS* (NRAS^Q61R^), but harboured no mutation in the other key components of the PI3K pathway, including *PIK3CA*, *PTEN,* and *PIB5PA* (Figure 1D) [31]. None of the melanoma cell lines harboured nonsynonymous mutations in the *INPP4B* gene as determined by sequencing all the 27 exons (including the intron/exon boundaries) of the gene. While INPP4B was expressed at undetectable or low levels in most of the melanoma cell lines (6/10), it was elevated in the others with varying levels (4/10) (Figure 1D). Noticeably, there was no significant relationship between INPP4B expression and the mutational status of BRAF or NRAS (Figure 1D and data not shown). Similar to the finding in fresh melanoma isolates, there was no association between INPP4B levels and Akt activation in melanoma cell lines (Figure 1D).

### INPP4B promotes proliferation of melanoma cells independently of Akt

**Figure 2:**
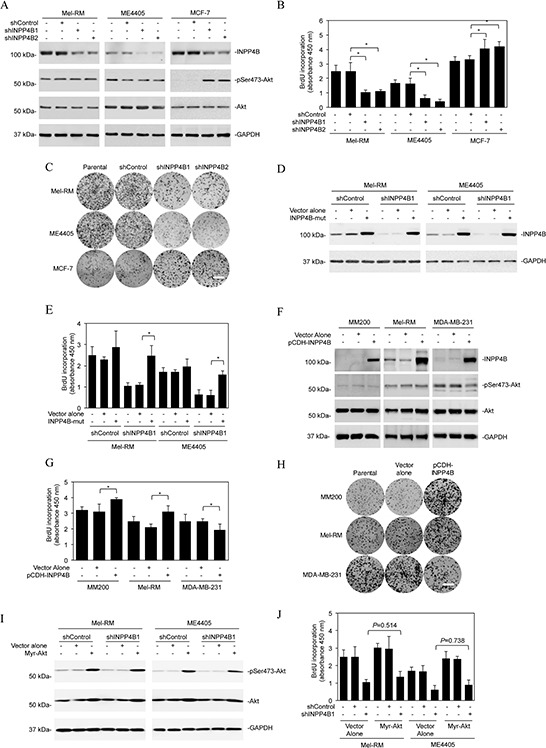
INPP4B promotes proliferation of melanoma cells independently of Akt **A.** Whole cell lysates from Mel-RM and ME4405 melanoma cells and MCF-7 breast cancer cells stably transduced with the control shRNA (shControl) or two INPP4B shRNAs (shINPP4B1 and shINPP4B2) were subjected to Western blot analysis of INPP4B, phosphorylated Akt (pSer473-Akt), Akt and GAPDH (as a loading control). The data shown are representative of three individual experiments. **B.** Mel-RM, ME4405 and MCF-7 cells were stably transduced with the control shRNA (shControl) or two INPP4B shRNAs (shINPP4B1 and shINPP4B2). Forty-eight hours later, cells were subjected to BrdU incorporation assays. The data shown are mean ± SEM of three individual experiments. *P < 0.05, Student's *t*-test. **C.** Mel-RM, ME4405 and MCF-7 cells stably transduced with the control shRNA (shControl) or two INPP4B shRNAs (shINPP4B1 and shINPP4B2) were subjected to clonogenic assays. The data shown are representative of three individual experiments. Scale bar, 1cm. **D.** Mel-RM and ME4405 cells stably transduced with shControl or shINPP4B1 were transduced with a shRNA-resistant mutant form of INPP4B (INPP4B-mut). Forty-eight hours later, whole-cell lysates were subjected to Western blot analysis. The data shown are representative of three individual experiments. **E.** Mel-RM and ME4405 cells stably transduced with shControl or shINPP4B1 were transduced with a shRNA-resistant mutant form of INPP4B (INPP4B-mut). Forty-eight hours later, cells were subjected to the BrdU incorporation assay. The data shown are mean ± SEM of three individual experiments. *P < 0.05, Student's *t*-test. **F.** Whole cell lysates from MM200 and Mel-RM melanoma cells and MDA-MB-231 breast cancer cells stably transduced with the vector alone or INPP4B cDNA cloned in the pCDH vector (pCDH-INPP4B) were subjected to Western blot analysis of INPP4B, phosphorylated Akt (pSer473-Akt), Akt and GAPDH (as a loading control). The data shown are representative of three individual Western blot analyses. **G.** MM200, Mel-RM and MDA-MB-231 cells stably transduced with the vector alone or INPP4B cDNA cloned in the pCDH vector (pCDH-INPP4B) were subjected to BrdU incorporation assays. The data shown are mean ± SEM of three individual experiments. *P < 0.05, Student's *t*-test. **H.** MM200, Mel-RM and MDA-MB-231 cells stably transduced with the vector alone or INPP4B cDNA cloned in the pCDH vector (pCDH-INPP4B) were subjected clonogenic assays. The data shown are representative of three individual experiments. Scale bar, 1cm. **I.** Mel-RM and ME4405 cells stably transduced with shControl or shINPP4B1 were transduced with the vector alone or myr-Akt cDNA. Forty-eight hours later, whole cell lysates were subjected to Western blot analysis of phosphorylated Akt (pSer473-Akt), Akt and GAPDH (as a loading control). The data shown are representative of three individual Western blot analyses. **J.** Mel-RM and ME4405 cells stably transduced with shControl or shINPP4B1 were transduced with the vector alone or myr-Akt cDNA. Forty-eight hours later, cells were subjected to BrdU incorporation assays. The data are mean ± SEM of three individual experiments.

We focused on examination of the functional significance of INPP4B upregulation in melanoma cells by knockdown of INPP4B with two individual shRNAs using lentiviral transduction in Mel-RM and ME4405 cells (Figure 2A). Surprisingly, INPP4B knockdown did not affect the basal levels of activation of Akt, nor did it enhance Akt activation triggered by stimulation with EGF (Supplementary Figure 2). Although INPP4B knockdown caused low levels of cell death, which was inhibited by the general caspase inhibitor z-VAD-fmk, indicative of apoptosis (Supplementary Figure 3) [37], inhibition of cell proliferation appeared to be the predominant functional consequence as shown by 5-bromo-2′-deoxyuridine (BrdU) incorporation and clonogenic assays (Figures 2B and 2C). Introduction of a construct expressing shRNA-resistant cDNA of INPP4B reversed the inhibitory effect of the INPP4B shRNAs on INPP4B expression and cell proliferation (Figures 2D and 2E), confirming the specificity of the INPP4B shRNAs. As anticipated and in contrast to its effect on melanoma cells, INPP4B knockdown enhanced Akt activation and promoted proliferation in MCF-7 cells that were used as a control (Figures 2A-C) [25, 26]. Collectively, these results suggest that, despite its tumour suppressive role mediated by inhibition of activation of Akt in MCF-7 cells, INPP4B promotes melanoma cell proliferation independently of activation of Akt. In support, introduction of exogenous INPP4B into MM200 cells that expressed low levels of INPP4B and Mel-RM cells led to, albeit moderately, increased cell proliferation, but did not alter the levels of Akt activation (Figures 2F-2H). In contrast, introduction of INPP4B into MDA-MB-231 breast cancer cells that similarly had low levels of endogenous INPP4B caused decreases in cell proliferation and Akt activation (Figures 2F-2H). Of note, although introduction of an active form of Akt (myr-Akt) promoted cell proliferation, it did not significantly reverse the inhibitory effect of INPP4B knockdown on proliferation of Mel-RM and ME4405 cells (Figures 2I-2J). Similarly, knockdown of Akt did not significantly reverse the promoting effect of INPP4B overexpression on Mel-RM and ME4405 cell proliferation (Supplementary Figure 4). These results consolidate the importance of INPP4B-mediated Akt-independent mechanism in melanoma cell proliferation.

### Activation of SGK3 plays an important role in INPP4B-mediated melanoma cell proliferation

**Figure 3:**
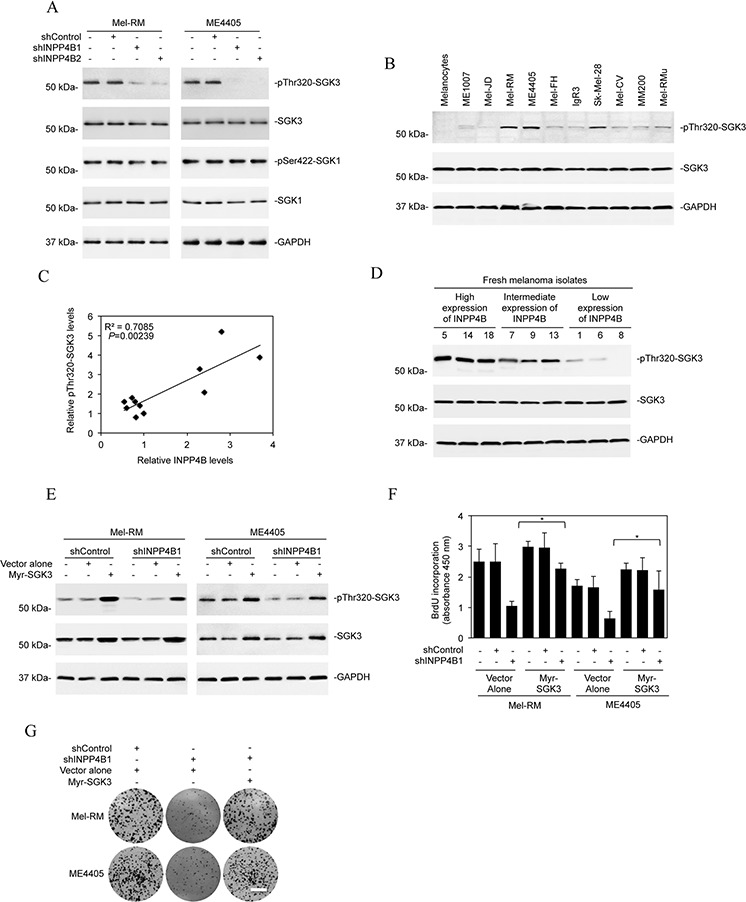
SGK3 is involved in INPP4B-mediated melanoma cell proliferation **A.** Whole cell lysates from Mel-RM and ME4405 cells stably transduced with the control shRNA (shControl) or INPP4B shRNA1 (shINPP4B1) were subjected to Western blot analysis of phosphorylated SGK3 (pThr320-SGK3), SGK3, phosphorylated SGK1 (pSer422-SGK1), SGK1, and GAPDH (as a loading control). The data shown are representative of three Western blot analyses. **B.** Whole cell lysates from the panel of melanoma cells lines and pooled melanocytes of three different lines (HEMa-LP, HEMn-MP and HEMn-DP) as shown in Figure 1D were subjected to Western blot analysis of phosphorylated SGK3 (pThr320-SGK3), SGK3 and GAPDH (as a loading control). The data shown are representative of three individual experiments. **C.** Regression analysis of the relationship between the levels of INPP4B expression as shown in Figure 1D and SGK3 activation as shown in Figure 3B. **D.** Whole-cell lysates from fresh melanoma isolates with relatively low, intermediate, and high levels of INPP4B were subjected to Western blot analysis of phosphorylated SGK3 (pThr320-SGK3), SGK3 and GAPDH (as a loading control). The data shown are representative of three individual Western blot analyses. **E.** Mel-RM and ME4405 cells stably transduced with the control shRNA (shControl) or INPP4B shRNA1 (shINPP4B1) were transduced with the vector alone or myr-SGK3 cDNA. Whole cell lysates were subjected to Western blot analysis of phosphorylated SGK3 (pThr320-SGK3), SGK3 and GAPDH (as a loading control). The data shown are representative of three individual Western blot analyses. **F.** Mel-RM and ME4405 cells stably transduced with the control shRNA (shControl) or INPP4B shRNA1 (shINPP4B1) were transduced with the vector alone or myr-SGK3 cDNA. Cells were subjected to BrdU incorporation assays. The data are mean ± SEM of three individual experiments. *P < 0.05, Student's *t*-test. **G.** Mel-RM and ME4405 cells stably transduced with the control shRNA (shControl) or INPP4B shRNA1 (shINPP4B1) were transduced with the vector alone or myr-SGK3 cDNA. Cells were subjected to clonogenic assays. The data shown are representative of three individual clonogenic assays. Scale bar, 1cm.

Since SGKs are highly homologous to and share substrate specificity with Akt [18, 20], we tested whether they are involved in INPP4B-mediated promotion of melanoma cell proliferation. Although shRNA knockdown of INPP4B did not impinge on phosphorylation of SGK1, it inhibited SGK3 activation in Mel-RM and ME4405 cells (Figure 3A), suggesting that INPP4B preferentially affects SGK3 activation in melanoma cells. Indeed, SGK3 activation (phosphorylation) was elevated in melanoma cell lines expressing relatively high levels of INPP4B compared with those with low levels (Figures 1D, 3B and 3C). In support, examination of representative fresh melanoma isolates with various levels of INPP4B showed that melanomas with high, intermediate, and low expression of INPP4B displayed progressively decreasing levels of phosphorylated (activated) SGK3 (Figure 3D). The functional significance of SGK3 was demonstrated by co-introduction of an active form of SGK3 (myr-SGK3) into Mel-RM and ME4405 cells with INPP4B stably knocked down, which, in contrast to myr-Akt, abolished suppression of proliferation resulting from knockdown of INPP4B (Figures 2I, 2J and 3E-3G). Collectively, these results indicate that INPP4B activates PI3K/SGK3 signalling to drive melanoma cell proliferation independently of Akt.

### INPP4B modulates melanoma growth

**Figure 4:**
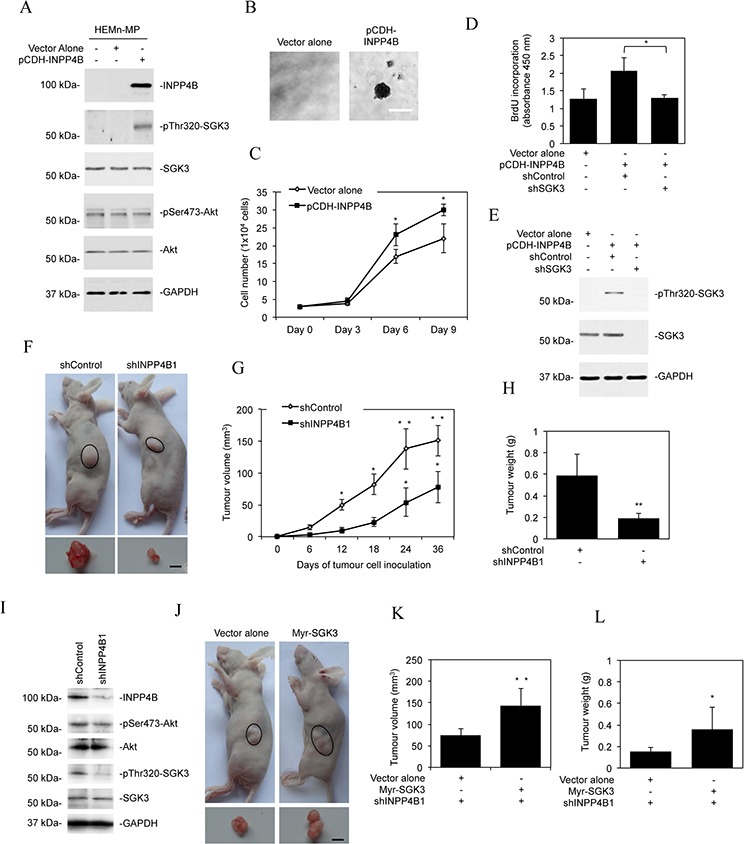
INPP4B promotes melanoma growth **A.** Whole cell lysates from HEMn-MP melanocytes stably transduced with the vector alone or INPP4B cDNA cloned in the pCDH vector (pCDH-INPP4B) were subjected to Western blot analysis of INPP4B, phosphorylated SGK3 (pThr320-SGK3), SGK3, phosphorylated Akt (pSer473-Akt), Akt and GAPDH (as a loading control). The data shown are representative of three individual Western blot analyses. **B.** Representative microphotographs of anchorage-independent growth of HEMn-MP melanocytes transduced with INPP4B cDNA cloned in the pCDH vector (pCDH-INPP4B). The data shown are representative of three individual clonogenic assays. Scale bar, 0.5 mm. **C.** Comparison of numbers of HEMn-MP melanocytes on day 3, 6, and 9 after being transduced with the vector alone or pCDH-INPP4B. The data shown are mean ± SEM of three individual experiments. *P < 0.05, Student's *t*-test. **D.** HEMn-MP melanocytes transduced with the vector alone or pCDH-INPP4B were transduced with the control shRNA (shControl) or SGK3 shRNA (shSGK3). Forty-eight hours later, cells were subjected to BrdU incorporation assays. The data shown are mean ± SEM of three individual experiments. *P < 0.05, Student's *t*-test. **E.** HEMn-MP melanocytes transduced with the or pCDH-INPP4B were transduced with the control shRNA (shControl) or SGK3 shRNA (shSGK3). Forty-eight hours later, whole-cell lysates were subjected to Western blot analysis of phosphorylated SGK3 (pThr320-SGK3), SGK3 and GAPDH (as a loading control). The data shown are representative of three individual experiments. **F.** Representative photographs showing that xenografts of Mel-RM cells transduced with the INPP4B shRNA1 (shINPP4B1) are smaller than those with the control shRNA (shControl) (*n* = 8). Scale bar, 5 mm. **G.** Comparison of growth curves of xenografts of Mel-RM cells transduced with the INPP4B shRNA1 (shINPP4B1) and those with the control shRNA (shControl). The data shown are mean ± SEM, *n* = 8. **P < 0.01, *P < 0.05 Student's *t*-test. **H.** Comparison of weight of harvested xenografts of Mel-RM cells transduced with the INPP4B shRNA1 (shINPP4B1) and those with the control shRNA (shControl). The data shown are mean ± SEM, *n* = 8. **P < 0.01, Student's *t*-test. **I.** Whole cell lysates of crude tumour tissues from representative harvested xenografts of Mel-RM cells transduced with the INPP4B shRNA1 (shINPP4B1) and those with the control shRNA (shControl) were subjected to Western blot analysis of INPP4B, phosphorylated Akt (pSer473-Akt), Akt, phosphorylated SGK3 (pThr320-SGK3), SGK3 and GAPDH (as a loading control). The data shown are representative of three individual Western blot analyses. **J.** Representative photographs showing that xenografts of Mel-RM cells transduced with the INPP4B shRNA1 (shINPP4B1) co-introduced with the cDNA encoding myr-SGK3 are larger than those co-introduced with the vector alone. (*n* = 8). Scale bar, 5 mm. **K.** Comparison of growth curves of xenografts of Mel-RM cells transduced with the INPP4B shRNA1 (shINPP4B1) co-introduced with the cDNA encoding myr-SGK3 and those co-introduced with the vector alone. The data shown are mean ± SEM, *n* = 8. **P < 0.01, Student's *t*-test. **L.** Comparison of weight of harvested xenografts of Mel-RM cells transduced with the INPP4B shRNA1 (shINPP4B1) co-introduced with cDNA encoding myr-SGK3 and those co-introduced with the vector alone. The data shown are mean ± SEM, *n* = 8. *P < 0.05, Student's *t*-test.

To study whether INPP4B-mediated activation of PI3K/SGK3 signalling plays a role in melanocyte transformation, we introduced an INPP4B-expressing construct into HEMn-MP melanocytes (Figure 4A). This triggered activation of SGK3 but not Akt, and caused anchorage-independent growth of the cells (Figures 4A and 4B). Similarly, INPP4B overexpression enhanced the proliferation potential of HEMn-MP melanocytes (Figure 4C), which could be reversed by co-introduction of a shRNA against SGK3, further substantiating the role of SGK3 in INPP4B-mediated proliferation of melanocytic cells (Figures 4D and 4E).

To examine whether the elevated expression of INPP4B affects melanoma growth *in vivo*, we transplanted Mel-RM cells with or without INPP4B stably knocked down into nu/nu mice. Deficiency in INPP4B caused significant retardation of tumour growth *in vivo* (Figure 4F-4H), which was associated with reduction in SGK3 activation (Figure 4I). The role of inhibition of SGK3 in suppression of melanoma xenograft growth was confirmed by transplanting INPP4B stable knockdown Mel-RM cells co-introduced with myr-SGK3 into nu/nu mice (Figures 3E and 4J-4L). This demonstrated that myr-SGK3 effectively restored tumour growth.

### Upregulation of INPP4B in melanoma cells is associated with loss of miR-494 and/or miR-599

**Figure 5:**
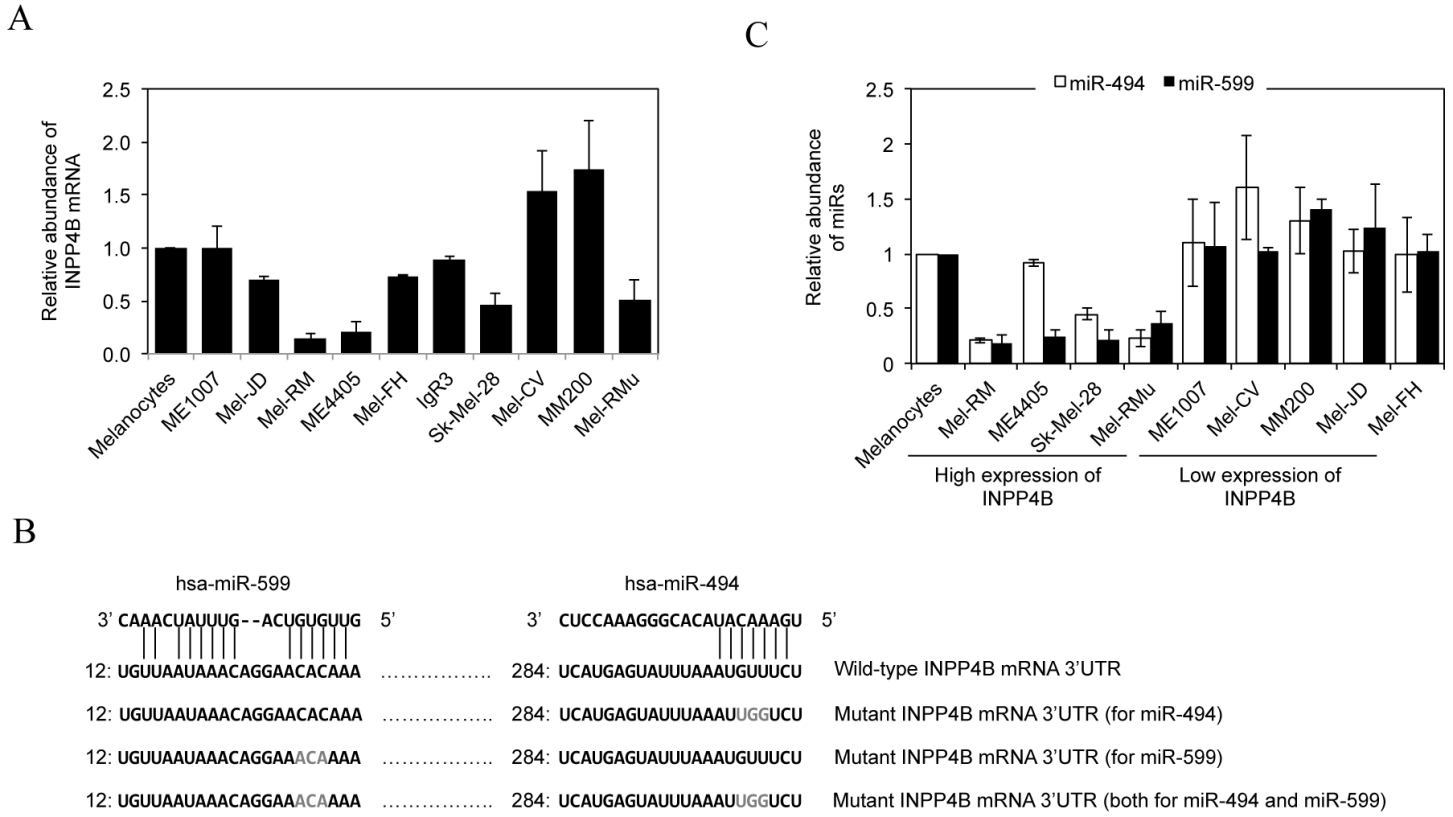
Upregulation of INPP4B in melanoma cells is associated with loss of miR-494 and/or miR-599 **A.** Total RNA from melanocytes and melanoma cells were subjected to qPCR analysis of INPP4B mRNA expression. The relative abundance of INPP4B mRNA in melanocytes was arbitrarily designated as 1. Data are represented as mean ± SEM of three individual experiments. **B.** A schematic illustration of base pairing between miR-494 and miR-599 and the 3′UTR of the INPP4B mRNA. Mutations of the binding regions for miR-494 and miR-599 at the INPP4B 3′UTR are also shown. **C.** Total RNA from HEMn-MP melanocytes and melanoma cells were subjected to qPCR analysis of miR-494 or miR-599 expression. The relative abundance of each miR in melanocytes was arbitrarily designated as 1. The data shown are mean ± SEM of three individual experiments.

To understand the mechanism responsible for INPP4B upregulation in melanoma cells, we quantitated the expression of INPP4B mRNA in the panel of melanoma cell lines in which the expression of INPP4B at the protein level has been characterized. The results showed that there were wide variations in INPP4B mRNA levels that were not correlated with the levels of the INPP4B protein (Figures 1D and 5A). For example, Mel-RM and ME4405 cells that had relatively high levels of the INPP4B protein expressed even relatively low levels of the INPP4B transcript (Figures 1D and 5A). These results suggest that INPP4B upregulation in melanoma cells is due to a posttranscriptional increase.

We examined whether miRNAs that commonly target transcripts to block their translation are involved in regulation of INPP4B by comparing miRNA expression profiles between melanoma cell lines expressing relatively high levels of the INPP4B protein (Mel-RM and ME4405) and those with low levels (MM200 and ME1007) using TaqMan^®^ Low Density miRNA Array [38]. Amongst miRNAs that were differentially expressed, reduction in miR-494 and miR-599 in Mel-RM cells and miR-599 in ME4405 cells were among the most pronounced (Supplementary Table 2). Intriguingly, the “seed” regions of miR-494 and miR-599 matched perfectly to regions at the 3′UTR of the INPP4B mRNA (http://www.microRNA.org; http://www.targetscan.org) (Figure 5B), suggesting that these miRs may target the INPP4B transcript. The differences in the expression of miR-494 and/or miR-599 between melanoma cell lines with different levels of the INPP4B protein were confirmed by qPCR (Figure 5C).

### Loss of miR-494 and/or miR-599 is responsible for upregulation of INPP4B

**Figure 6:**
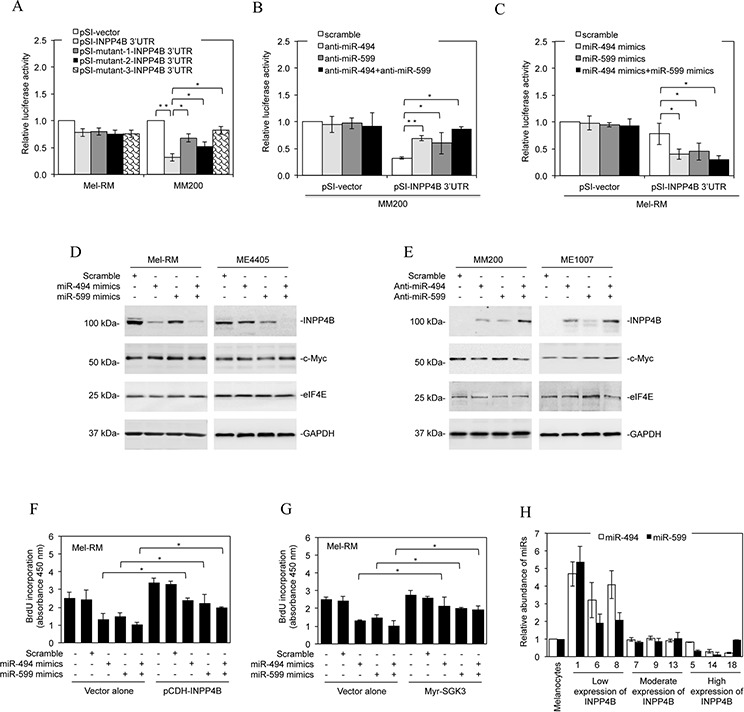
Downregulation of miR-494 and miR-599 contributes to upregulation of INPP4B **A.** Mel-RM and MM200 cells were co-transfected with the indicated reporter constructs and luciferase plasmids. Twenty-four hours later, the reporter activity was measured using luciferase assays. Fold-activation values were measured relative to the levels of Renilla luciferase activity in cells transfected with the vector alone (pSI-vector) and normalized by Firefly luciferase activities. The data shown are the mean ± SEM of three individual experiments. **P < 0.01, *P < 0.05, Student's *t*-test. **B.** MM200 cells were co-transfected with the indicated reporter constructs and Renilla luciferase plasmids. Scrambled, anti-miR-494, anti-miR-599, or anti-miR-494 plus anti-miR-599 oligonucleotides were co-transfected. Twenty-four hours later, the reporter activity was measured using luciferase assays. Fold-activation values were measured relative to the levels of Renilla luciferase activity in cells transfected with the vector alone (pSI-vector) and scrambled oligonucleotides (scramble) and normalized by Firefly luciferase activities. The data shown are the mean ± SEM of three individual experiments. **P < 0.01, *P < 0.05, Student's *t*-test. **C.** Mel-RM cells were transfected with the indicated reporter constructs. Scrambled, miR-494 mimics, miR-599 mimics, or miR-494 mimics plus miR-599 mimics were also co-transfected. Twenty-four hours later, the reporter activity was measured using luciferase assays. Fold-activation values were measured relative to the levels of Renilla luciferase activity in cells transfected with the vector alone (pSI-vector) and scrambled oligonucleotides (scramble) and normalized by Firefly luciferase activities. The data shown are the mean ± SEM of three individual experiments. *P < 0.05, Student's *t*-test. **D.** Mel-RM and ME4405 cells were transfected with scrambled, miR-494 mimics, miR-599 mimics, or miR-494 mimics plus miR-599 mimics. Twenty-four hours later, whole cell lysates were subjected to Western blot analysis of INPP4B, c-Myc, eIF4E and GAPDH (as a loading control). Data shown are representative of three individual experiments. **E.** MM200 and ME1007 cells were transfected with scrambled, anti-miR-494, anti-miR-599, or anti-miR-494 plus anti-miR-599 oligonucleotides. Twenty-four hours later, whole cell lysates were subjected to Western blot analysis of INPP4B, c-Myc, eIF4E and GAPDH (as a loading control). Data shown are representative of three individual experiments. **F.** Mel-RM cells were co-transfected with scrambled, miR-494 mimics, miR-599 mimics, or miR-494 mimics plus miR-599 mimics oligonucleotides along with the vector alone or cDNA encoding INPP4B (pCDH-INPP4B). Forty-eight hours later, cells were then subjected to BrdU incorporation assays. The data shown are the mean ± SEM of three individual experiments. *P < 0.05, Student's *t*-test. **G.** Mel-RM cells were co-transfected with scrambled, miR-494 mimics, miR-599 mimics, or miR-494 mimics plus miR-599 mimics oligonucleotides along with the vector alone or cDNA encoding myr-SGK3. Forty-eight hours later, cells were then subjected to BrdU incorporation assays. The data shown are the mean ± SEM of three individual experiments. *P < 0.05, Student's *t*-test. **H.** qPCR analysis of miR-494 and miR-599 in total RNA from HEMn-MP melanocytes and fresh melanoma isolates sampled according to the levels of INPP4B protein as shown in Figure 1C. The relative abundance of miR-494 or miR-599 in melanocytes was arbitrarily designated as 1. The data shown are the mean ± SEM of three individual experiments.

To test whether miR-494 and miR-599 target INPP4B, we introduced luciferase reporter plasmids of the 3′UTR of INPP4B into Mel-RM and MM200 cells (Figure 5B and Supplementary Figure 5A). PCR analysis confirmed that the fragment of DNA cloned into the luciferase reporter plasmids was indeed present at the endogenous 3′UTR of INPP4B in melanoma cells (Supplementary Figure 5B). The reporter activity was markedly suppressed by the presence of the 3′UTR of INPP4B in MM200 but not in Mel-RM cells. However, this suppression was partially reversed when the binding region for miR-494, and to a lesser extent, the binding region for miR-599, was mutated (Figures 5B, 6A and Supplementary Figure 5A). When the binding regions for miR-494 and miR-599 were mutated simultaneously, there was a further increase in the promoter activity (Figures 5B, 6A and Supplementary Figure 5A), suggesting that miR-494 and miR-599 function corporately to target the 3′UTR of INPP4B in melanoma cells. In support, co-introduction of anti-miR-494 and anti-miR-599 into MM200 cells increased the reporter activity to a greater extent than introduction of either of the anti-miRNA alone (Figure 6B and Supplementary Figures 6A and B). On the other hand, co-introduction of miR-494 and miR-599 mimics concurrently resulted in further reduction in the reporter activity in comparison with introduction of either miR-494 or miR-599 mimics alone (Figure 6C and Supplementary Figures 6C and D).

The functional significance of miR-494 or miR-599 in supressing INPP4B expression was consolidated by downregulation of endogenous INPP4B with introduction of miR-494 or miR-599 mimics into Mel-RM and ME4405 cells, whereas co-introduction of miR-494 and miR-599 mimics caused further reduction in INPP4B (Figure 6D). On the other hand, introduction of anti-miR-494 or anti-miR-599 into MM200 and ME1007 cells upregulated the expression of endogenous INPP4B, whereas co-introduction of anti-miR-494 and anti-miR-599 resulted in a further increase (Figure 6E). Noticeably, neither anti-miR-494 nor anti-miR-599 or the combination of both impinges on the expression levels of co-introduced exogenous INPP4B that lacks the 3′UTR region (Supplementary Figure 7). These results substantiate that INPP4B is a *bona fide* target of miR-494 and miR-599 in melanoma cells. The endogenous levels of another predicted miR-494 targets, c-Myc, and another predicted miR-599 targets, eIF4E, were not affected by introduction of miR-494 mimics and miR-599 mimics, respectively, in Mel-RM and ME4405 cells (Figure 6D). Similarly, c-Myc and eIF4E were not affected by introduction of anti-miR-494 and anti-miR-599 in MM200 and ME1007 cells, respectively (Figure 6E). Therefore, upregulation of INPP4B through loss of miR-494 and miR-599 is highly selective in melanoma cells. Introduction of miR-494 or miR-599 mimics, and to a greater extent, co-introduction of both, caused inhibition of proliferation in Mel-RM cells, which was abolished by co-introduction of exogenous INPP4B or myr-SGK3 (Figures 6F and 6G), consolidating the functional significance of downregulation of these miRNAs in melanoma cells.

We also examined representative fresh melanoma isolates sampled by their INPP4B protein levels for the expression of miR-494 and miR-599. The results showed that melanomas with no or low expression of INPP4B displayed relatively high levels of miR-494 and miR-599, whereas those expressing relatively high levels of INPP4B exhibited reduction in miR-494 and/or miR-599 (Figures 1C and 6H). Therefore, the inhibitory effect of miR-494 and miR-599 on INPP4B *in vivo* is in accordance with the regulatory model identified *in vitro*.

### Loss of miR-494 and miR-599 is associated with gene copy number reduction

**Figure 7:**
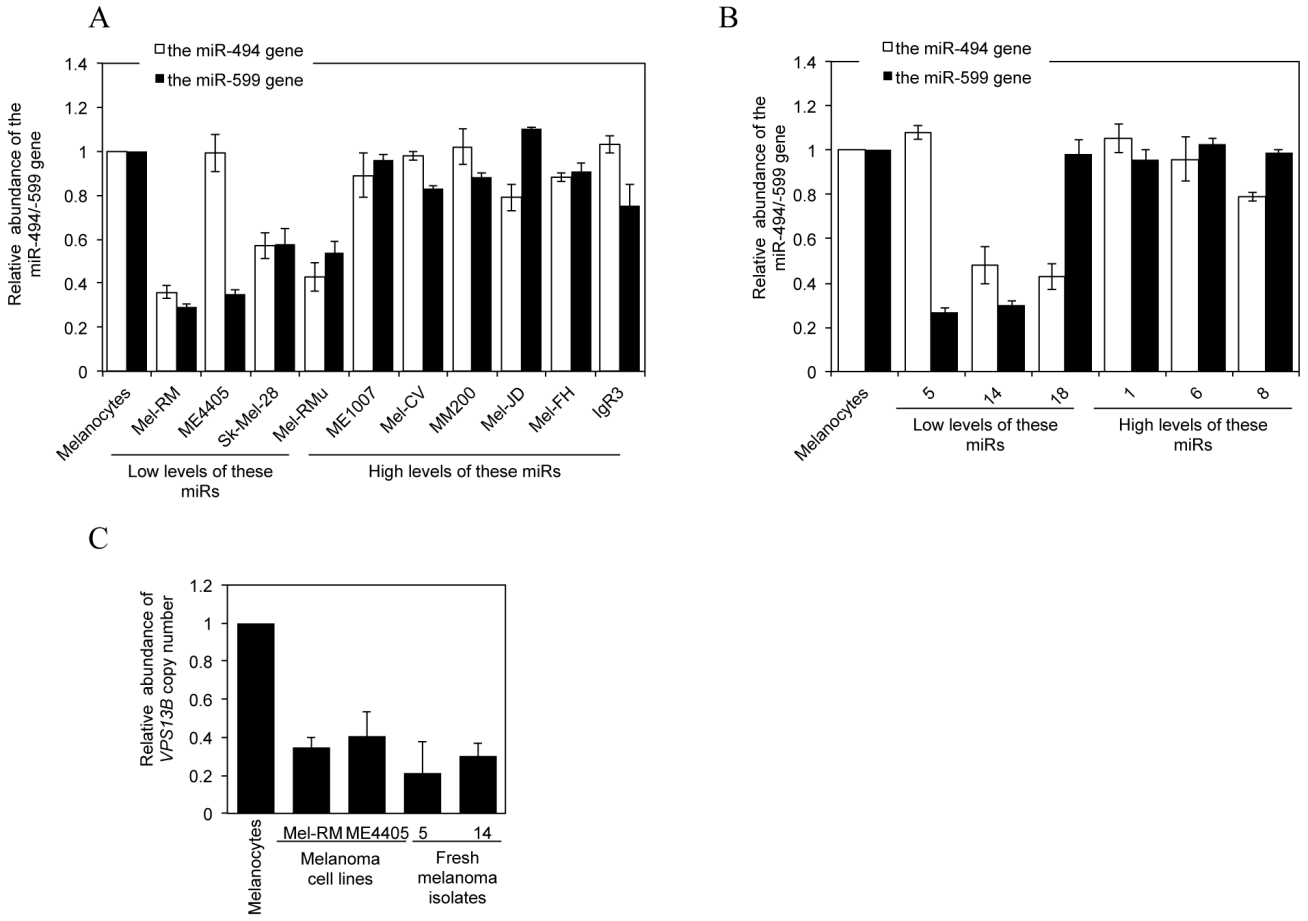
Downregulation of miR-494 or miR-599 in melanoma cells is associated with DNA copy number reduction **A.** qPCR analysis of genomic DNA from HEMn-MP melanocytes and melanoma cells. The average copy number of each gene in melanocytes was arbitrarily designated as 1. The data shown are the mean ± SEM of three individual experiments. **B.** qPCR analysis of genomic DNA from HEMn-MP melanocytes and fresh melanoma isolates sampled according to the levels of INPP4B protein as shown in Figure 1C. The average copy number of each gene in melanocytes was arbitrarily designated as 1. The data shown are the mean ± SEM of three individual experiments. **C.** qPCR analysis of genomic DNA from HEMn-MP melanocytes, Mel-RM and ME4405 melanoma cells and fresh melanoma isolates. The average copy number of melanocytes was arbitrarily designated as 1. The data shown are the mean ± SEM of three individual experiments.

MiR-encoding genes are frequently located to clusters at fragile sites in the genome where genomic alterations frequently take place [39, 40]. We therefore examined whether loss of miR-494 and miR-599 in melanoma cells is associated with gene copy number variations. qPCR analysis of genomic DNA revealed that copy number reduction of one or both of the genes encoding these miRs was found in the subsets of melanoma cell lines and fresh melanoma isolates that displayed decreased expression of these miRNAs (Figures 7A and 7B). As anticipated, the copy number of the host gene of miR-599 *VPS13B* is similarly reduced in melanoma cells and fresh melanoma isolates with low levels of miR-599 (Figure 7C).

## DISCUSSION

In this report, we present evidence that the 4-phosphatase INPP4B has an unexpected oncogenic role in human melanoma. While INPP4B was upregulated in a subset of melanomas, knockdown of INPP4B caused reduction in SGK3 activation, which led to inhibition of proliferation of melanoma cells *in vitro* and retardation in melanoma growth in a xenograft model. On the other hand, introduction of exogenous INPP4B resulted in increased melanoma cell and melanocyte proliferation and anchorage-independent growth of melanocytes. Our results also revealed that loss of miR-494 and miR-599 due to gene copy number reduction was responsible for upregulation of INPP4B in melanoma cells.

INPP4B is emerging as a tumour suppressor in various types of cancers including prostate cancer, ovary cancer, and triple-negative breast cancer [25, 26, 32, 41]. While the tumour suppressive role of INPP4B in these tissues has been exclusively attributed to its ability to inhibit PI3K/Akt signalling, INPP4B did not appear to impinge on Akt activation in melanoma cells and melanocytes. This is surprising, since INPP4B has been well-demonstrated to dephosphorylate PI(3,4)P_2_ at the 4-position, thus inhibiting PI3K/Akt activation [25, 26]. Nevertheless, although both PI(3,4)P_2_ and PI(3,4,5)P_3_ can recruit and activate Akt, a number of studies have shown that PI(3,4,5)P_3_ is more important for Akt activation [27, 42–44]. It is likely that activation of Akt is predominantly mediated by PI(3,4,5)P_3_, and that the negative effect of INPP4B on PI(3,4)P_2_ does not significantly affect Akt activation in melanocytic cells. Although this needs to be further investigated, our results clearly point to an Akt-independent mechanism downstream of PI3K that is responsible for INPP4B-mediated proliferation of melanocytic cells. By analogue, INPP4B is known to activate SGK3 and drive tumourigenesis in a subset of breast cancers with low Akt [34]. Nevertheless, there was no significant difference in the expression levels of Akt across the melanoma cell lines and fresh melanoma isolates. This suggests that the preferential effect of INPP4B on SGK3 is not related to reduced Akt expression, but is dictated by the cell type-dependent functional consequence of INPP4B in melanocytic cells. Full activation of SGK3 requires its translocation to early endosomes [18, 20], which is mediated by binding of SGK3 to PI(3)P through its N-terminal PX domain [18, 20]. INPP4B-mediated PI(3,4)P_2_ dephosphorylation at the 4 position generates PI(3)P [26, 32]. This may contribute to the increased activation of SGK3. Nevertheless, as the most abundant phosphatidylinositol phosphate in cells [45], PI(3)P is primarily generated by phosphorylation of phosphatidylinositol (PI) catalyzed by class III, and to a lesser extent, class II PI3K [45, 46]. Whether INPP4B is involved in regulation of the biogenesis of PI(3)P by class III and II PI3Ks in melanocytic cells needs to be clarified.

Although Akt is the best-characterized downstream effector of PI3K signalling, PI3K can mediate cell proliferation and survival independently of Akt through activation of SGKs, in particular, SGK1 and SGK3 [18, 20]. Indeed, SGK3 contributes to the pathogenesis of various malignant tumours such as breast and ovary cancer and hepatocellular carcinoma [18, 20]. In particular, a recent report demonstrated an important role of SGK3 in promoting mutant BRAF melanoma growth [23]. Our results extended this observation by showing that SGK3 similarly played a role in proliferation of wild-type BRAF melanoma cells (Mel-RM and ME4405) and melanocytes, at least when the upstream signal was driven by INPP4B. Although the downstream effectors responsible for SGK3-mediated melanoma cell proliferation remain to be studied, it is conceivable that regulators of cell proliferation such as p27 and p21 that can be directly or indirectly regulated by Akt are involved, in that SGK3 shares substrate specificities with Akt [19, 20].

An important finding of this study is the identification of loss of miR-494 and miR-599 as the mechanism responsible for upregulation of INPP4B in melanoma cells. As a fine-tune mechanism to regulate gene expression, a miRNA commonly targets 3′ UTRs of a group of mRNAs [47, 48]. On the other hand, the 3′ UTR of a mRNA is usually targeted by more than one miRNA [47, 48]. Indeed, we found that miR-494 and miR-599 cooperatively suppressed INPP4B in melanoma cells, which were nevertheless lost either individually or concurrently in a proportion of melanomas. Given that melanoma cells are highly heterogeneous, it is conceivable that the relative importance of the two miRs in suppression of INPP4B may also be highly variable depending on cells and context in question.

Consistent with the frequent localization of miR-encoding genes to clusters at fragile sites in the genome where genomic alterations frequently take place [39, 40], copy number reduction of the genes encoding miR-494 and/or miR-599 was found in melanoma cell lines and fresh melanoma isolates with low levels of these miRs. MiR-494 is located to a segment of chromosome 14q32.31, whereas the host gene of miR-599 *VPS13B* is located to segment of chromosome 8q22.2. Whether reduction in copy number of the genes encoding for the miRs are due to genomic variations at extended fragments of chromosome remains to be determined, but the copy number of *VPS13B* is similarly reduced in melanoma cell lines and fresh isolates with low levels of miR-599. Moreover, if other mechanisms such as epigenetic regulation are involved in suppression of these miRs in melanoma cells needs to be clarified [49, 50].

In summary, we have identified INPP4B as an oncogenic driver that promotes melanoma cell proliferation independently of Akt through activation of PI3K/SGK3 signalling, and revealed loss of miR-494 and miR-599 due to gene copy number reduction as the mechanism responsible for upregulation of INPP4B in a subset of melanomas (Supplementary Figure 8). However, whether elevated INPP4B is associated with resistance to treatment and poor patient outcome in melanoma as is it in AML remains to be clarified [35, 36]. Similarly, whether INPP4B is targetable in the treatment of melanoma needs further investigation, but given that INPP4B functions as a tumour suppressor in many other tissues [25, 26, 32, 41], direct inhibition of INPP4B *in vivo* needs to be evaluated with a great caution. Alternatively, suppression of INPP4B indirectly through restoring miR-494 and miR-599 may be a useful strategy, which may lead to selective inhibition of INPP4B in melanoma cells, as regulation of target expression by miRs is known to be highly cell type-dependent [51–53].

## MATERIALS AND METHODS

### Ethics statement

Investigation using human tissues and animals has been conducted in accordance with the ethical standards according to national and international guidelines. Studies using human tissues were approved by the Human Research Ethics Committees of the University of Newcastle and Royal Prince Alfred Hospital, Australia. Studies on animals were approved by the Animal Research Ethics Committee of Shanxi Cancer Hospital, China.

### Cell culture and human tissues

The human melanoma cell lines used have been described previously [31]. They were cultured in DMEM containing 5% FCS (Commonwealth Serum Laboratories, Melbourne, VIC, Australia). The melanocyte lines HEMa-LP, HEMn-MP and HEMn-DP and melanocyte culture medium (M-254) were purchased from Banksia Scientific (Bulimba QLD, Australia). Human fresh melanoma isolates were prepared as described before [31]. Tissue microarrays (TMAs) were constructed from formalin-fixed paraffin-embedded melanocytic tumour tissues retrieved from the Department of Tissue Pathology and Diagnostic Oncology at the Royal Prince Alfred Hospital, Australia (Supplementary Table S1).

### Antibodies and reagents

Antibodies against Akt, phospho-Akt (Ser473) and phospho-SGK3 (Thr320) were from Cell Signalling Technology (Beverly, MA). Antibodies against INPP4B, SGK3, phospho-SGK1 (Ser422), c-Myc and eIF4E were purchased from Santa Cruz Biotechnology (Santa Cruz, CA). Antibody against SGK1 was from Millipore (Billerica, MA). Mature hsa-miR-494 or -599 mimics and anti-hsa-miR-494 or -599 were purchased from Life Technologies Australia Pty Ltd (VIC, Australia).

### IHC

IHC staining was performed on a Dako autostainer (Dako, Denmark) as described previously [31]. The INPP4B antibody specificity was confirmed by pre-absorption with recombinant full-length INPP4B that abolished its immunoreactivity in IHC assays.

### Immunoblotting

Immunoblotting was carried out as described previously [31]. The intensity of the bands was quantified using the NIH Image J.

### Cell viability

Cell viability was quantiated using the CellTiter-Glo Luminescent Cell Viability Assay kit according to the manufacturer's instructions (Promega, San Luis Obispo, CA). Luminescence was recorded by Synergy 2 multi-detection microplate reader (BioTek, VT).

### BrdU proliferation assays

BrdU cell proliferation assays were carried out using the BrdU Cell Proliferation Assay kit (Cell Signalling) as per the manufacturer's instructions as described previously [31].

### Clonogenic assays

Cells were seeded at 2000 cells/well onto 6-well culture plates. Cells were then allowed to grow for a further 12 days before fixation with methanol and staining with crystal violet (0.5% solution).

### Anchorage-independent cell growth

5 × 10^4^ melanocytes HEMn-MP cells transduced with vector alone or INPP4B cDNA were seeded in 0.3% cell agar layer, which was on top of 0.6% base agar layer in 12-well culture plates. Cells were then incubated for a further 30 days at 37°C and 5% CO_2_. Cell colony formation was then examined under a light microscope.

### Melanoma xenograft mouse model

Mel-RM cells with INPP4B stably knocked down and those transduced with the control shRNA with or without co-introduced with myr-SGK3 were injected subcutaneously into each flank of male athymic nude mice (Model Animal Research Centre of Nanjing University, China). Each experimental group consisted of 8 mice. Tumour growth was monitored as previously described [31].

### Short hairpin RNA (shRNA)

MISSION^®^ human shRNA lentiviral transduction particles: INPP4B (TRCN0000230837 and TRCN0000230838), SGK3 (TRCN0000199867) as well as the corresponding control particles were purchased from Sigma-Aldrich. These shRNAs were used to infect cells according to the manufacturer's protocol.

### Quantitative reverse transcription-PCR (qPCR) analysis of mRNA expression

qPCR analysis of mRNA expression was performed was performed as described previously [31]. The primer sequences for INPP4B are: forward, 5′-CCC CGG GTA CTG AGG CTT CG-3′, reverse, 5′-CTT TGT ATT CTC TCC CGG AGG CG-3′.

### qPCR analysis of copy number variations

Genomic DNA was extracted with the Wizard^®^ Genomic DNA Purification Kit according to manufacturer's instructions (Promega). The specific primers used to were: for the miR-494 gene, forward: GGA GAG GTT GTC CGT GTT GT; reverse: GGC TGC ATC AGG AAC AGG AA; for the miR-599 gene, forward: CCT GGG ACC CCA TTA TCC TT; reverse: TGC TGT CCA CAG TGT GTT TG; for *VPS13B*, forward: TGT TCC AGA TGG AGC CTT TC; reverse: CAT GAT TCC TTT CCC CAC AC. As a control, the housekeeping gene *HBB* was also amplified and quantitated. The primers for *HBB* were: forward: 5′-ACA CAA CTG TGT TCA CTA GC-3′; reverse: 5′-CAA CTT CAT CCA CGT TCA CC-3′.

### qPCR analysis of miRNA

Quantitation of miR-494 or -599 by qPCR was performed using TaqMan^®^ microRNA assays according to manufacturer's instruction (Life Technologies Australia Pty Ltd.) and described previously [38].

### TaqMan^®^ low density array (TLDA) miR array

miRNA array was performed using TaqMan^®^ TLDA card according to manufacturer's instruction (Life Technologies Australia Pty Ltd.) using an ABI Fast 7900HT sequence detection system (Life Technologies Australia Pty Ltd.) as described previously [38]. Relative microRNA abundance was calculated with the RQ Manager v1.2.1, and data was analyzed with DataAssist v2.0 (Life Technologies Australia Pty Ltd.). The expression levels of miRs were calculated against the Ct value of RNU48 as 2 ^(−ΔCt)^ / 2^(−ΔCtreference)^.

### Lentiviral gene transduction and DNA constructs

The *INPP4B*, myr-*SGK3* or myr-*Akt* were cloned into the lentiviral expression plasmid pCDH-CMV-MCS-EF1-copGFP (Integrated Sciences, Chatswood, NSW, Australia). A mutant INPP4B construct containing four silent mutations within the INPP4B shRNA1 target sequence was also cloned into the pCDH-CMV-MCS-EF1-copGFP plasmid. Production of recombinant lentiviruses and infection of cells were carried out as described before [54].

### Luciferase reporter assays

Luciferase report assays to analyze INPP4B-3′UTR activity was performed as described previously [38, 55]. The luciferase activity was measured using the Dual Luciferase Reporter Assay System (Promega, Alexandria, NSW, Australia) and detected by Synergy 2 multi-detection microplate reader (BioTek, VT).

### Statistical analysis

Statistical analysis was performed using GraphPad Prism (La Jolla, CA). Student's *t*-test or Kruskal-Wallis test was used to assess differences in the expression of the proteins between different groups. A *P* value less than 0.05 was considered statistically significant.

## SUPPLEMENTARY FIGURES AND TABLES


