# Supplementary material for: INPP4B is upregulated and functions as an oncogenic driver through SGK3 in a subset of melanomas
Source: Oncotarget. 2015 Nov 9;6(37):39891–907. doi: 10.18632/oncotarget.5359 (PMC4741868; doi:10.18632/oncotarget.5359)
Supplement: Supplementary file 1 [file oncotarget-06-39891-s001.pdf]

SUPPLEMENTARY FIGURES AND TABLES

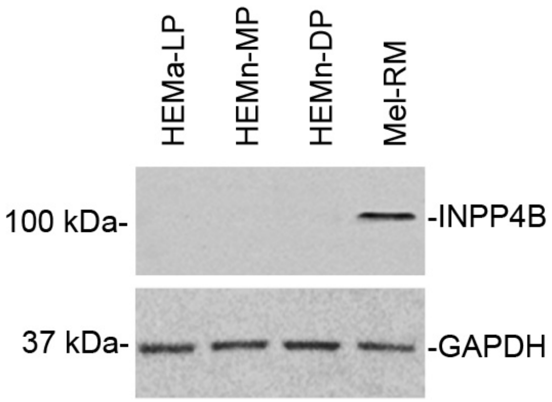

Supplementary Figure S1: Melanocyte lines of different origins express similarly undetectable levels of INPP4B. Whole cell lysates were subjected to Western blot analysis of INPP4B and GAPDH (as a loading control). Data shown are representative of three individual experiments.

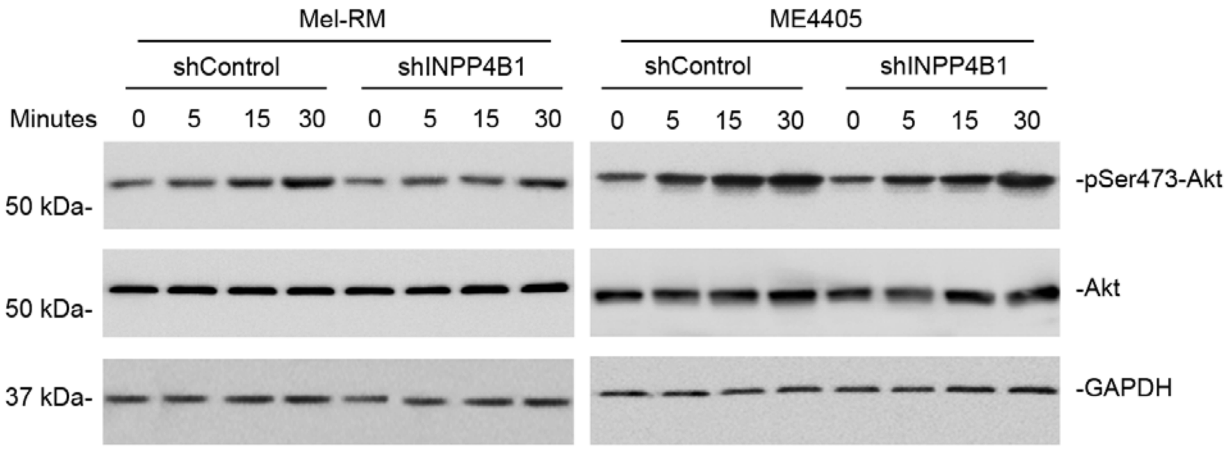

Supplementary Figure S2: INPP4B knockdown does not enhance Akt activation triggered by stimulation with EGF. Mel-RM and ME4405 cells stably transduced with the control shRNA (shControl) or INPP4B shRNA (shINPP4B1) were serum-starved for 16 hours before stimulation with EGF (100 nM) for indicated periods. Whole cell lysates were subjected to Western blot analysis of phosphorylated Akt (pSer473-Akt), Akt and GAPDH (as a loading control). The data shown are representative of three individual experiments.

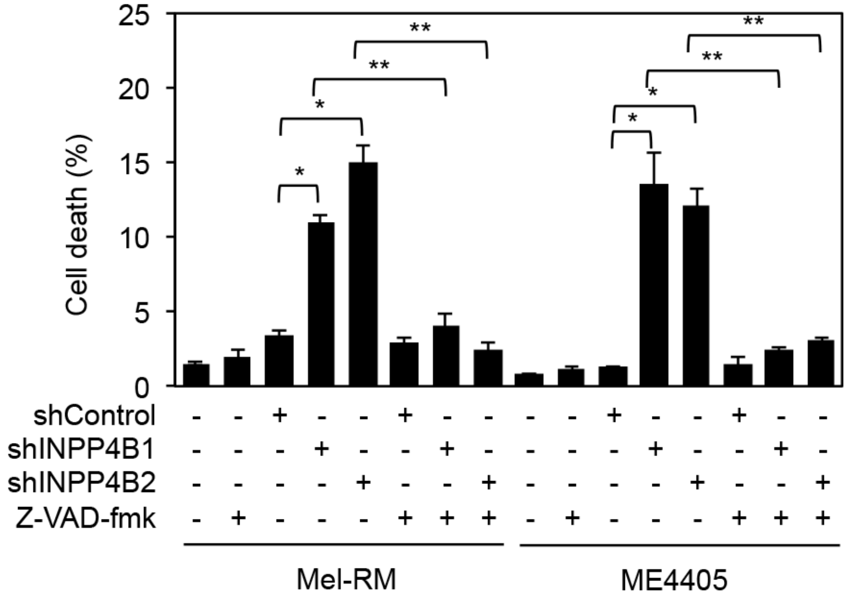

Supplementary Figure S3: INPP4B knockdown triggers low levels of apoptosis. Mel-RM and ME4405 cells transiently transduced with shControl, shINPP4B1, or shINPP4B2 were cultured in medium containing the general caspase inhibitor z-VAD-fmk. Fourty-eight hours later, cells were subjected to CellTiter-Glo assays. Data are represented as mean ± SEM of three individual experiments. $**P < 0.01$, $*P < 0.05$, Student's *t*-test.

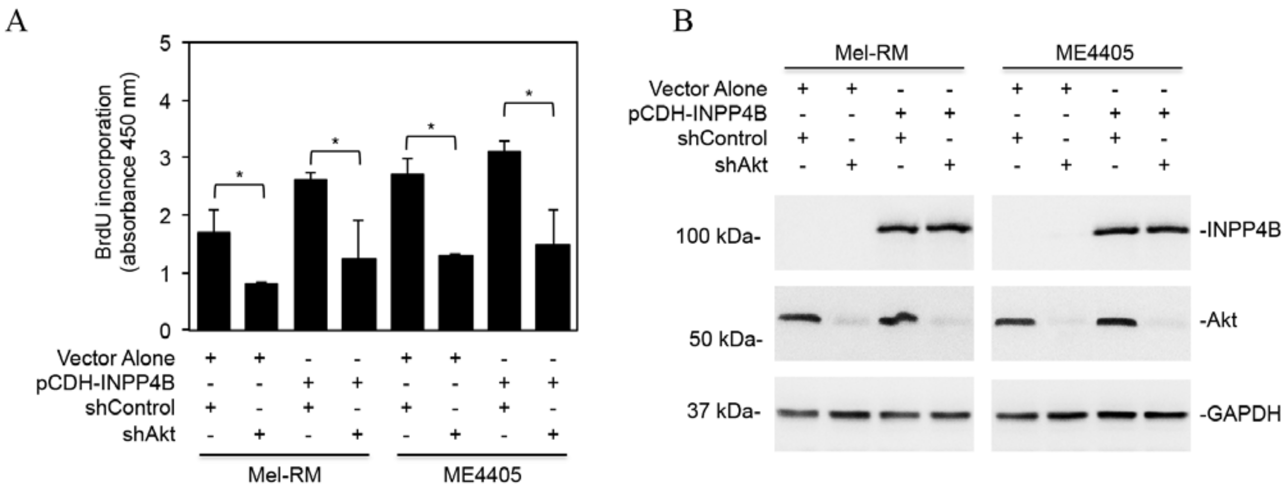

Supplementary Figure S4: Knockdown of Akt does not significantly reverse the promoting effect of INPP4B overexpression on melanoma cell proliferation. A. Mel-RM and ME4405 cells stably transduced with the vector alone or INPP4B cDNA cloned in the pCDH vector (pCDH-INPP4B) were transiently transduced with the Control shRNA (shControl) or Akt shRNA (shAkt). Forty-eight hours later, cells were then subjected to BrdU incorporation assays. The data shown are mean ± SEM of three individual experiments. *$P < 0.05$, Student's *t*-test. **B.** Mel-RM and ME4405 cells stably transduced with the vector alone or INPP4B cDNA cloned in the pCDH vector (pCDH-INPP4B) were transiently transduced with Control shRNA (shControl) or Akt shRNA (shAkt). Forty-eight hours later, whole cell lysates were subjected to Western blot analysis of INPP4B, Akt and GAPDH (as a loading control). Data shown are representative of three individual experiments.

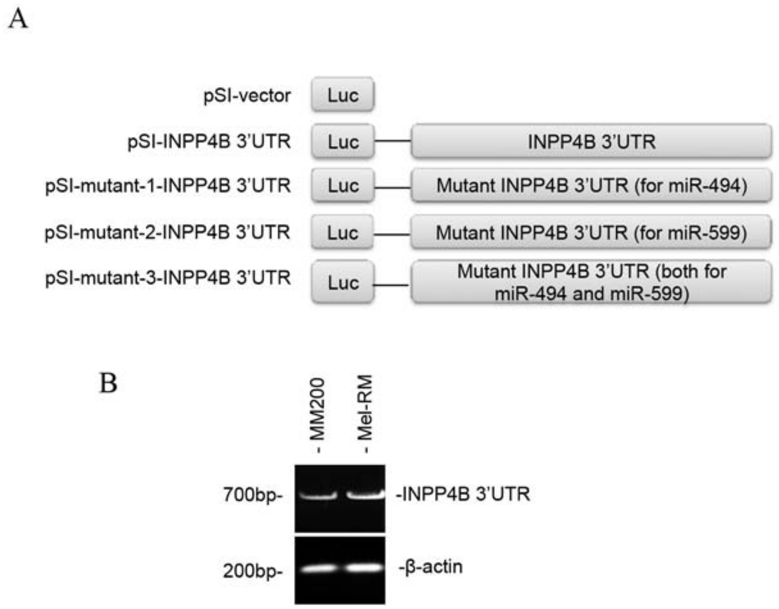

Supplementary Figure S5: A. Schematic illustrations of the pSI-CHECK2 luciferase reporter constructs used for examining the effects of miR-494 and miR-599 on the INPP4B 3′UTR. Constructs with the INPP4B 3′UTR mutated at the predicted miR-494 or miR-599 binding site are also depicted. **B.** RT-PCR analysis showing that the fragment of INPP4B mRNA that was cloned into the luciferase reporter plasmids was present in MM200 and Mel-RM cells. Total mRNA of MM200 and Mel-RM cells was subjected to PCR analysis of the INPP4B 3′UTR. The data shown are representative of three individual experiments.

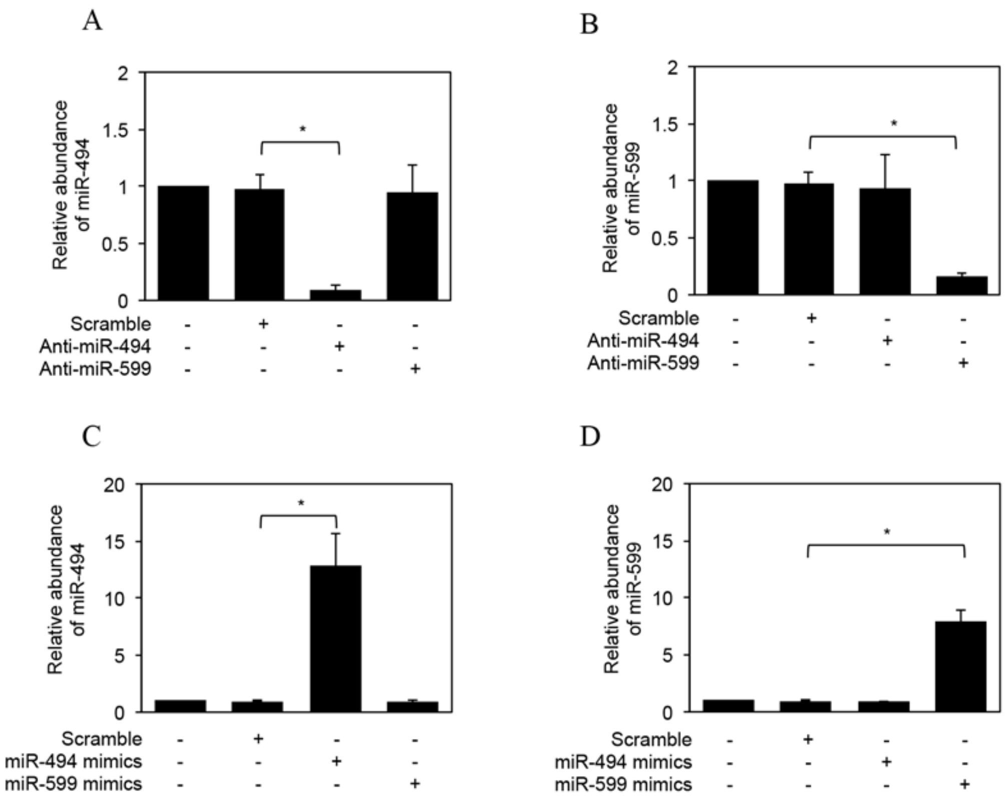

Supplementary Figure S6: The effect of anti-miR-494, anti-miR-599, miR-494 mimcs, or miR-599 mimics on miR-494 or miR-599 expression. **A** and **B.** qPCR analysis of miR-494 (A), or miR-599 (B) in total mRNA from MM200 cells transfected with scrambled, anti-miR-494 or anti-miR-599 oligonucleotides. The relative abundance of miR-494 (A) or miR-599 (B) in cells transfected with scrambled oligonucleotides (scramble) was arbitrarily designated as 1. The data shown are the mean ± SEM of three individual experiments. *P <0.05, Student's t-test. **C** and **D.** qPCR analysis of miR-494 (C) or miR-599 (D) in total mRNA from Mel-RM cells transfected with scrambled, miR-494 mimics or miR-599 mimics. The relative abundance of miR-494 (C) or miR-599 (D) in cells transfected with scrambled oligonucleotides (scramble) was arbitrarily designated as 1. The data shown are the mean ± SEM of three individual experiments. *P <0.05, Student's t-test.

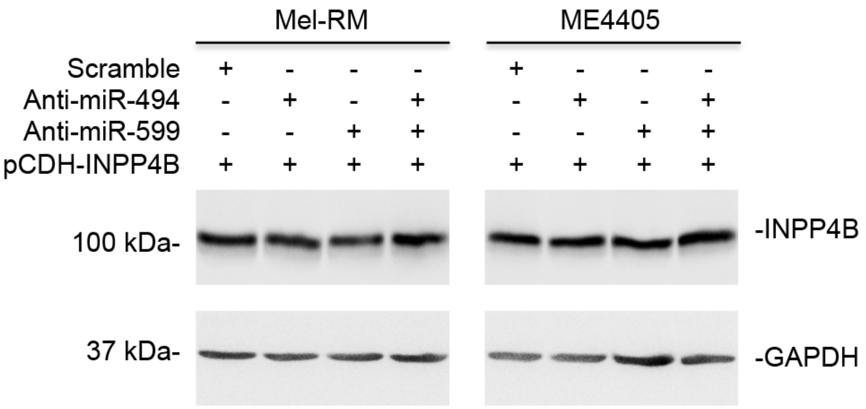

Supplementary Figure S7: Inhibition of miR-494 and/or miR-599 does not impinge on the exogenous INPP4B level. MM200 and ME1007 cells stably transduced with the vector alone or INPP4B cDNA cloned in the pCDH vector (pCDH-INPP4B) were transfected with scrambled, anti-miR-494, anti-miR-599, or anti-miR-494 plus anti-miR-599 oligonucleotides. Twenty-four hours later, whole cell lysates were subjected to Western blot analysis of INPP4B and GAPDH (as a loading control). Data shown are representative of three individual experiments.

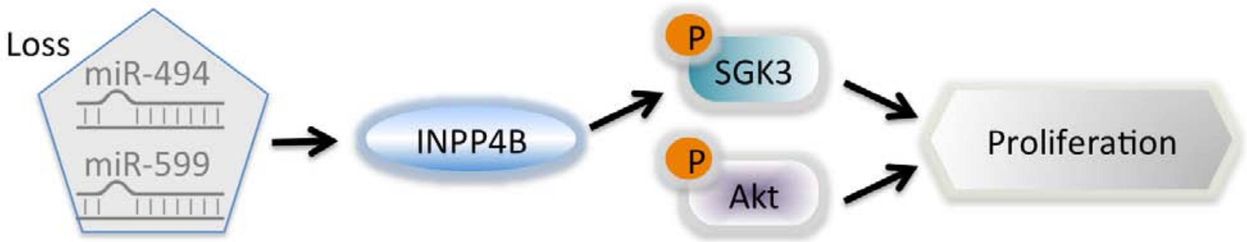

Supplementary Figure S8: A Schematic illustration of the mechanism responsible for regulation of PI3K/SGK3 signalling by the increased expression INPP4B as a result of loss of miR-494 and/or miR-599 in melanoma cells. It is of note that INPP4B promotes melanoma cell through activation of PI3K/SGK3 signalling proliferation independently of Akt.

Supplementary Table S1: Summary of melanocytic tumors and their positivity for INPP4B

Melanocytic tumors	Number	% INPP4B positivemelanoma cells
Compound Nevus	10	67.3[1] (58–90)[2]
Dysplastic Nevus	10	72.4 (63–95)
Thin Primary (<1mm Breslow depth)	20	96.8 (94–98)
Thick Primary (>1mm Breslow depth)	20	98.2 (96–100)
Lymph Node Metastases	20	92.4 (85–100)
Distant Metastases	20	97.5 (88–100)

[1]Numbers stand for the mean percentage of positive cells
[2]Numbers in brackets represent the range of positive cells.

Supplementary Table S2: List of miRs that are commonly increased or decreased for at least 1.5 folds in melanoma cell lines compared to melanocytes[1]

	MM200	ME1007	Mel-RM	ME4405
hsa-miR-148a	11.184[2]	18.435	−2.589[3]	−2.39
hsa-miR-574–3p	2.0389	4.2032	−2.214	−6.46
hsa-miR-615–5p	3.7014	46.863	−2.079	−4.16
hsa-miR-627	3.9757	2.4336	−1.858	−1.644
hsa-miR-494	6.7909	12.528	−4.615	−1.511
hsa-miR-599	5.5106	6.2767	−3.624	−8.354

[1]The level of a miRNA in melanocytes was arbitrarily designated as 1
[2]Numbers stand for folds of changes in melanoma cell lines
[3]- represents reduction in expression.
